# Visible to Infrared Diamond Photonics Enabled by Focused Femtosecond Laser Pulses

**DOI:** 10.3390/mi8020060

**Published:** 2017-02-17

**Authors:** Belén Sotillo, Vibhav Bharadwaj, John Patrick Hadden, Stefano Rampini, Andrea Chiappini, Toney T. Fernandez, Cristina Armellini, Ali Serpengüzel, Maurizio Ferrari, Paul E. Barclay, Roberta Ramponi, Shane M. Eaton

**Affiliations:** 1 Department of Physics, Politecnico di Milano, Piazza Leonardo da Vinci 32, Milano 20133, Italy; bsotillo@gmail.com (B.S.); vibhavbharadwaj@gmail.com (V.B.); toney.teddyfernandez@gmail.com (T.T.F.); roberta.ramponi@polimi.it (R.R.); 2 Istituto di Fotonica e Nanotecnologie-Consiglio Nazionale delle Ricerche (IFN-CNR), Piazza Leonardo da Vinci 32, Milano 20133, Italy; stefano.rampini@gmail.com; 3 Institute for Quantum Science and Technology, University of Calgary, Calgary, AB T2N 1N4, Canada; jpe.hadden@gmail.com (J.P.H.); pbarclay@ucalgary.ca (P.E.B.); 4 Institute of Photonics and Nanotechnology of the National Research Council (IFN-CNR), Characterization and Development of Materials for Photonics and Optoelectronics (CSMFO) and The Centre for Materials and Microsystems (FBK-CMM), Trento 38123, Italy; andrea.chiappini@unitn.it (A.C.); cristina.armellini@ifn.cnr.it (C.A.); maurizio.ferrari@unitn.it (M.F.); 5 Microphotonics Research Laboratory, Department of Physics, Koç University, Rumelifeneri Yolu, Istanbul 34450, Turkey; aserpenguzel@ku.edu.tr

**Keywords:** diamond, nitrogen-vacancy (NV) center, femtosecond laser, laser micromachining, optical waveguide, magnetometry

## Abstract

Diamond’s nitrogen-vacancy (NV) centers show great promise in sensing applications and quantum computing due to their long electron spin coherence time and because they can be found, manipulated, and read out optically. An important step forward for diamond photonics would be connecting multiple diamond NVs together using optical waveguides. However, the inertness of diamond is a significant hurdle for the fabrication of integrated optics similar to those that revolutionized silicon photonics. In this work, we show the fabrication of optical waveguides in diamond, enabled by focused femtosecond high repetition rate laser pulses. By optimizing the geometry of the waveguide, we obtain single mode waveguides from the visible to the infrared. Additionally, we show the laser writing of individual NV centers within the bulk of diamond. We use µ-Raman spectroscopy to gain better insight on the stress and the refractive index profile of the optical waveguides. Using optically detected magnetic resonance and confocal photoluminescence characterization, high quality NV properties are observed in waveguides formed in various grades of diamond, making them promising for applications such as magnetometry, quantum information systems, and evanescent field sensors.

## 1. Introduction

Apart from its remarkable beauty when cut appropriately, diamond is the hardest naturally occurring bulk material, has a record high thermal conductivity and offers outstanding transparency from the ultraviolet to far infrared. However, it is actually a defect in diamond’s tetrahedral lattice of carbon atoms which has scientists excited. In both naturally found and synthetically fabricated diamond, the nitrogen-vacancy (NV) center is present, where a nitrogen sits next to an empty site in the carbon lattice. The optically active spin defects boasts long room temperature spin coherence time [1], making them attractive for efficient nanoscale magnetic field sensing and for quantum information systems.

An integrated optics platform in diamond would be beneficial for magnetometry, due to the enhanced interaction provided by waveguides, and quantum computing, in which NV centers could be optically linked together for long-range quantum entanglement. However, despite some previous attempts [2,3], it remains a challenge to fabricate optical waveguides in diamond due to its hardness and chemical inertness.

Recently, a disruptive technology based on femtosecond laser microfabrication was proposed to enable a 3D photonics toolkit for diamond [4,5]. Since focused ultrashort laser pulses damage the crystalline lattice [6], an indirect approach was applied: optical mode confinement was achieved between two closely spaced and parallel laser-inscribed modification lines. We discuss the mechanisms for waveguiding using this type II fabrication method and the role of the repetition rate. We show for the first time the fabrication of optical waveguides in ultrapure diamond that can address single NV centers in quantum information systems. In addition, we show shallow waveguide formation in the less pure high-pressure high-temperature (HPHT) diamond, of relevance for magnetometry. 

For practical implementation of quantum information systems, an important requirement is the deterministic placement of NVs. The common method to place NV centers in diamond relies on ion implantation techniques to insert single ions of nitrogen [7]. Although this method offers submicron spatial accuracy, it has limitations with respect to the depth that NVs can be created. Recently, on-demand single NVs in the bulk of ultrapure diamond were demonstrated using static femtosecond laser exposures, aided by a spatial light modulator (SLM) to correct for spherical aberrations and produce symmetric modifications [8]. In the present work, we demonstrate the laser writing of single NVs in quantum grade diamond without the use of special beam shaping techniques. The ability to write waveguide devices that exploit diamond NV centers would be compelling for quantum information and spin-based sensors, as it would enable novel 3D device configurations for interacting with NVs. 

## 2. Materials and Methods 

The femtosecond laser used for optical waveguide writing in diamond was a regeneratively amplified Yb:KGW system (Pharos, Light Conversion, Vilnius, Lithuania) with 230-fs pulse duration, 515-nm wavelength (frequency doubled), focused with a 1.25-NA oil immersion lens (RMS100X-O 100× Plan Achromat Oil Immersion Objective, Olympus, Tokyo, Japan). The repetition rate of the laser was variable from 500 kHz to single pulse. Computer-controlled, 3-axis motion stages (ABL-1000, Aerotech, Pittsburgh, PA, USA) were used to translate the sample relative to the laser to form the optical circuits in synthetic diamond. 

Polished synthetic single-crystal HPHT (3 mm × 3 mm × 0.3 mm, nitrogen impurities 100 ppm), optical grade (5 mm × 5 mm × 0.5 mm, type II, nitrogen impurities 100 ppb), and quantum grade (dimensions 2 mm × 2 mm × 0.3 mm, type II, nitrogen impurities <5 ppb) diamond samples were acquired from MB Optics (Velp, Netherlands). 

For waveguide transmission measurements, high-resolution 3-axis manual positioners (Nanomax MAX313D, Thorlabs, Newton, NJ, USA) were used. The four-axis central waveguide stage (MicroBlock MBT401D, Thorlabs) enabled transverse displacement between sets of diamond waveguides. Light sources at 1550 nm (TLS001-1550, Thorlabs), 808 nm (S1FC808, Thorlabs), 635 nm (TLS001-635, Thorlabs), and 532 nm (4301-010, Uniphase) were coupled to the waveguides using the Thorlabs single-mode fibers (P1-SMF28E-FC-2 for 1550 nm, 780HP for 808 nm, SM600 for 635 nm, and 460HP for 532 nm). At the output, light was coupled to an optical power meter (818-IG-L or 818-SL, Newport, Irvine, CA, USA) to measure the power transmitted through the waveguide. To measure the near-field waveguide mode profile, a 60× asphere (5721-H-B, Newport) was used to image the light to a charge-coupled device (CCD) (SP620U, Spiricon, North Logan, UT, USA).

Micro-Raman spectra were recorded using a Labram Aramis Jobin Yvon Horiba microRaman system with a DPSS laser source of 532 nm and equipped with a confocal microscope and an air-cooled CCD. A 100× objective was used to focus the laser on the sample as well as to collect the Raman signal, with a spatial resolution of about 1 µm. A wavenumber accuracy of about 1 cm^−1^ can be achieved with a 1800 line/mm grating. Photoluminescence measurements were also done with this system using a 600 line/mm grating.

For confocal photoluminescence measurements, nitrogen-vacancy defects were excited with a DPSS 532-nm laser (CL532-500-L, CrystaLaser, Reno, NV, USA) focused on to the sample with a 0.8 NA objective (100× CFI60 TU Plan Epi ELWD, Nikon, Tokyo, Japan). Photoluminescence was collected through the same objective, filtered from the excitation light using a dichroic beamsplitter (ZT 532 RDC, Chroma, Bellow Falls, VT, USA) and long-pass filters (ET 555 LP Chroma, FELH 0650 Thorlabs) and focused into a single mode fiber which provided the confocal aperture. Photon counting of the filtered light was performed using an avalanche photodiode (SPQR-14, Perkin-Elmer, Waltham, MA, USA). 

Annealing of the diamond samples was performed in a tubular horizontal furnace Lenton LTF15/50/450. The samples were placed in a quartz boat and covered with diamond grit to protect the surface. In order to purge the furnace chamber, oxygen was extracted using a diaphragm pump and a nitrogen flow for 1 h. Thermal treatments were carried out in a nitrogen atmosphere following three steps: first, temperature was raised to 1000 °C in 3 h and then kept at 1000 °C for another 3 h, and the furnace was finally switched off and allowed to cool down to room temperature. 

## 3. Results and Discussion

### 3.1 Optical Waveguides

**Figure 1:**
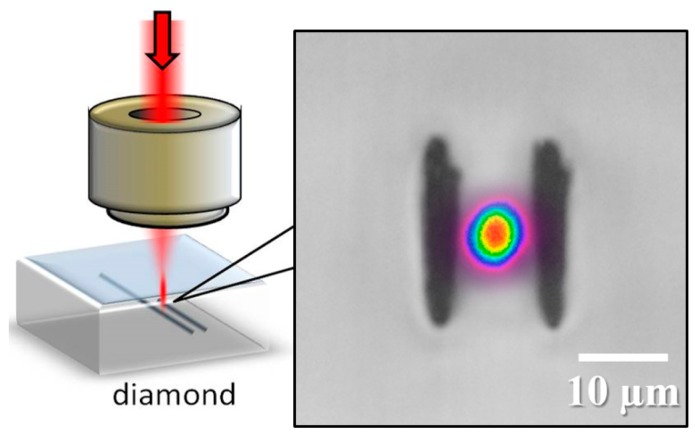
(**Left**) Type II waveguide geometry in diamond where optical mode confinement is achieved between two closely spaced and parallel laser-inscribed modification lines. (**Right**) Transverse view optical microscope image of type II modification in diamond, with modification tracks separated by 13 µm. The laser processing conditions were 500 kHz repetition rate, 100 nJ pulse energy, and 0.5 mm/s scan speed. The depth of the waveguide track (measured from surface to center of modifications) was 40 µm. Overlaid mode profile measured at 635 nm wavelength. Guiding could also be observed by translating the input fiber, a few microns above and below this central and highest transmission mode. Note that the color linear scale varying from 0 (violet) to 1 (red) indicates the relative intensity of the mode profile, normalized to the peak intensity.

Previously, we demonstrated that high repetition rates (~500 kHz) were favorable for avoiding graphite during femtosecond laser modification of diamond [4]. The highest repetition rate of 500 kHz available from our laser was thus applied to write two closely spaced modification lines, which yielded the first demonstration of optical waveguiding in diamond using femtosecond laser inscription (Figure 1). Using a low 5 kHz repetition rate, we could not detect optical waveguiding between the closely spaced modifications tracks. We attributed the lower damping losses at 500 kHz to the lower amount of graphite formed, as confirmed by µ-Raman spectroscopy and absorption measurements.

In parallel, a group from Oxford demonstrated that optical waveguides could indeed be formed in diamond using a low (1 kHz) repetition rate [5]. Using an SLM to produce more symmetric modifications compared to our highly elliptical modifications (Figure 1), the authors used a gentler interaction with lower energy pulses (30 nJ) to modify the bulk of diamond. To achieve optical waveguiding, however, a multiscan writing geometry was required with 6 symmetrical modifications spaced 3 µm apart vertically, to achieve similar elliptical confinement regions as our work. It is possible that the lower fluence interaction resulted in reduced graphite formation at this low repetition rate, yielding optical waveguides with reasonably low damping loss. 

The lowest measured insertion loss for our type II waveguides is 11 dB including 1.4 dB/facet coupling loss (0.5-cm sample length). The insertion loss is lower than previously reported due to the use of a PM fiber to launch TM polarized light into the waveguides. 

#### 3.1.1 µ-Raman Spectroscopy and Photoluminescence in the Waveguiding Region

**Figure 2:**
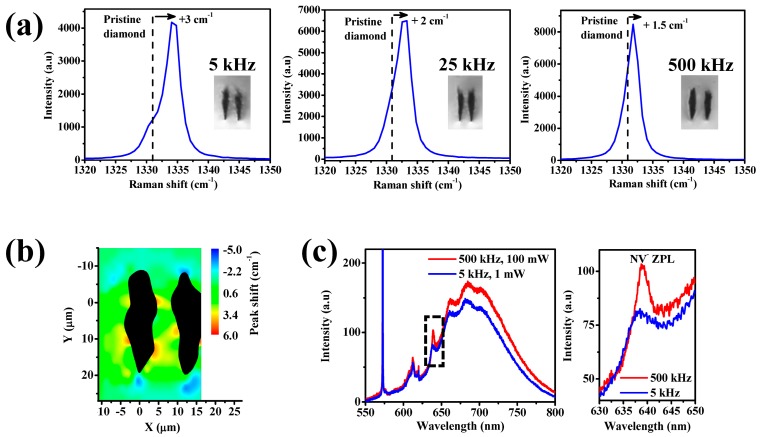
Effect of the repetition rate on the stress and the photoluminescence (PL) of the nitrogen-vacancy (NV) centers for double-line waveguides. (**a**) Shift of the Raman diamond peak at the center of the waveguide for different repetition rates. (**b**) Map of the shift of the Raman peak in a waveguide fabricated with 5 kHz. The laser modification tracks, where the amorphous carbon phase is formed, are shown as black. (**c**) PL of the NVs recorded at the center of waveguides fabricated with 500 kHz and 5 kHz repetition rates. The inset shows a zoomed in view of the spectra near the zero phonon line (ZPL) transition.

The repetition rate used not only affects the graphite formation inside the modification tracks, but also the quality of the crystal in the guiding region. To study this effect, we wrote double-line structures at 5, 25, and 500 kHz repetition rates, keeping other parameters constant (0.5 mm/s scan speed, 200 nJ pulse energy, 13 µm separation between lines, 40 µm depth) to obtain similarly sized modification tracks (Figure 2a). In µ-Raman spectroscopy, a shift of the diamond peak to higher (lower) wavenumber is associated with a compressive (tensile) stress. Mapping the shift of the diamond peak around the laser-modified lines gives information about the spatial distribution and type of stress that is produced in the guiding region.

The µ-Raman spectra in the guiding regions revealed a shift of the diamond peak towards higher wavenumbers, an indication of compressive stress. As shown in Figure 2a, the compressive stress is greater for lower repetition rates. Previous studies have shown that compressive stress in diamond results in a decrease of the refractive index [9], which is detrimental for optical waveguiding. The mechanisms for waveguiding in diamond are still under investigation, but could be the result of increased polarizability [10], a reduced refractive index in the modification lines, or loss-guiding quantum effects [11].

At 500 kHz repetition rate, the peak width is about 2 cm^−1^ and similar to the pristine value, showing that the crystalline structure is preserved. However, this peak width increases as the repetition rate is lowered, reaching a value of 3 cm^−1^ at 5 kHz repetition rate, which indicates a slight disordering of the crystal. Further evidence of this disorder in the diamond crystal lattice is the decrease in the Raman intensity for lower repetitions rates. 

A closer inspection of the Raman peak shows that it is not only shifted but also split into two components (one located at the position of the pristine peak, the second one at higher wavenumber). This effect is most evident at the lowest 5 kHz repetition rate. This split is associated with the presence of biaxial or uniaxial stress between the two damage lines [12] and could explain why only the TM polarization of light can be detected in our diamond waveguides.

The stress in the guiding region in not only higher for the lower repetition rates but also more nonuniform, as shown in the Raman shift map of Figure 2b. The shift observed for the guiding region formed with 5 kHz repetition rate varies from 1 to 4 cm^−1^, whereas for 500 kHz repetition rate the shift shows a uniform value of about 1.5 cm^−1^ [4]. 

Therefore, the higher losses observed for lower repetition rates could be due to several effects: increased graphite formation within the modification lines, higher compressive stress leading to less optical confinement and increased disorder in the guiding region. The good quality of the diamond that we have observed for 500 kHz may be associated with increased heat accumulation that is achieved for higher repetition rates [13]. 

The quality of the diamond crystal can affect the properties of the NV centers that can be found in the guiding region. Having this in mind, we have measured the photoluminescence at room temperature of the NVs at the center of the guiding region of the structures formed with 5 kHz and with 500 kHz repetition rates. The excitation wavelength was 532 nm, and the PL spectra are presented in Figure 2c. For 500 kHz, the zero phonon line (ZPL) of the NVs remains similar to that of pristine diamond (not shown), whereas for 5 kHz the ZPL is broader and its intensity is decreased. We attribute this broadening of the ZPL to the higher stress and disorder [14] in the guiding region for a 5 kHz repetition rate. Therefore, the elevated repetition rate of 500 kHz is not only beneficial for waveguiding but also for preserving the functionality of the NV centers. 

#### 3.1.2 Shallow Waveguides for Sensing and Magnetometry

The type II waveguide in Figure 1 exhibited multiple coupling locations vertically within the guiding region. To achieve coupling to a single mode at only one location, we used a four line type II modification, where tracks were also written above and below the guiding region shown in Figure 1. In the present work, we explored a different range of waveguide depths, with emphasis on shallower waveguides, which can interact with near-surface NV centers for applications in magnetometry.

**Figure 3:**
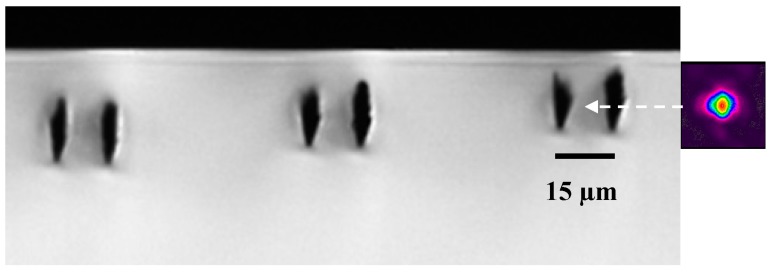
Side view optical microscope image of type II modification in diamond, with modification tracks separated by 15 µm. The laser processing conditions were 500 kHz repetition rate, 60 nJ pulse energy, and 0.5 mm/s scan speed. The waveguide depths (left to right) were and 20, 15, and 12 µm. Coupling to a single vertical position was possible with depths below 30 µm, with the minimum depth being 12 µm for waveguide operation. Mode profile at 635 nm characterization wavelength shown for the shallowest waveguide. Note that the color linear scale varying from 0 (violet) to 1 (red) indicates the relative intensity of the mode profile, normalized to the peak intensity. The 15 µm scale bar is the same for the mode and microscope image.

We found that for depths shallower than 30 µm, the two line type II modifications revealed single mode behavior at only a single, central location. We attribute this transition to single mode guiding at shallower depths to the reduced spherical aberration, which results in less vertically elongated modifications. As a result, the guiding region has a less vertical extent, allowing for the coupling to only a single vertical location. For waveguides characterized at visible wavelengths (532–800 nm), we found the minimum waveguide depth to be ~12 µm, as shown in Figure 3. Depths shallower than 12 µm resulted in surface laser ablation and very high insertion losses.

**Figure 4:**
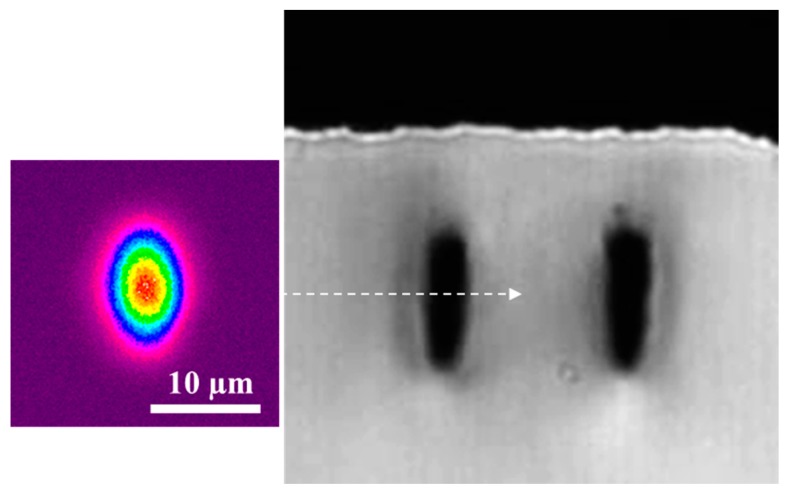
Side view of type II waveguide for 1550 nm guiding. The separation between the modification tracks was 19 µm and the depth was 20 µm. The laser processing conditions were 500 kHz repetition rate, 60 nJ pulse energy, and 0.5 mm/s scan speed. Single mode guiding was obtained at 1550 nm. Note that the color linear scale varying from 0 (violet) to 1 (red) indicates the relative intensity of the mode profile, normalized to the peak intensity. The 10 µm scale bar is the same for the mode and microscope image.

We also explored a range of separation distances between the modification tracks for extending the guiding capabilities to longer wavelengths, namely 1550 nm, which is of interest for evanescent coupling to surface photonics such as microspheres [15,16]. For this longer wavelength, the optimum separation distance was 19 µm, for which we achieved single mode operation (Figure 4). Similar waveguide loss and mode profile were found at 1550 nm compared to waveguides optimized for visible wavelength operation. 

All of the above reported waveguide results were achieved in optical grade diamond. In ultrapure quantum grade diamond and lower purity HPHT diamond, similar transmission characteristics were observed for the same laser processing parameters. Waveguides operational in quantum grade and HPHT diamond will be beneficial for optical devices in quantum information systems and magnetometry, respectively. 

### 3.2. NV Centers in Quantum Grade Diamond

For practical implementation of a diamond-based magnetometer or quantum information system, an important requirement is the deterministic placement of NV defects, while preserving the NV’s excellent spin properties. Applying the same femtosecond laser to fabricate both the optical waveguides and NV centers would enable a true diamond photonics fabrication platform, ensuring excellent alignment of NVs with optical circuitry. 

It has been shown previously that a femtosecond laser can create color centers in various materials [17,18,19]. In the case of diamond, we have seen from photoluminescence measurements within the modification lines [4] that the femtosecond laser creates amorphous carbon as well as a large amount of defects, which are mainly vacancies and vacancy complexes. Therefore, by using a single focused femtosecond laser pulse using an energy below the amorphization threshold, it may be possible to produce vacancies within the focal volume. In quantum grade diamond, there are up to 1000 nitrogen impurities present within the ~1 µm^3^ focal volume of the focused femtosecond laser beam, so the probability of creating a vacancy next to an impurity is low. The sample is subsequently annealed in order to create NV centers. Vacancies, which become mobile above the temperature of 600 °C are captured by the substitutional nitrogen impurities to form NV centers [20]. Higher temperature annealing may be used to anneal out other vacancy complexes, which can be detrimental to the NV centers’ properties [21].

This strategy was recently applied successfully by Chen et al. [8], using single static femtosecond laser pulses focused in the bulk of ultrapure quantum grade diamond. A SLM was applied by the researchers to achieve a symmetric modification, resulting in the formation of single NVs after annealing. Here we show that single NVs can be produced without spatial beam shaping methods. Using the same 1.25-NA oil immersion microscope objective applied for optical waveguide writing, we found that single NVs could be formed in quantum grade diamond at depths less than 30 µm. 

**Figure 5:**
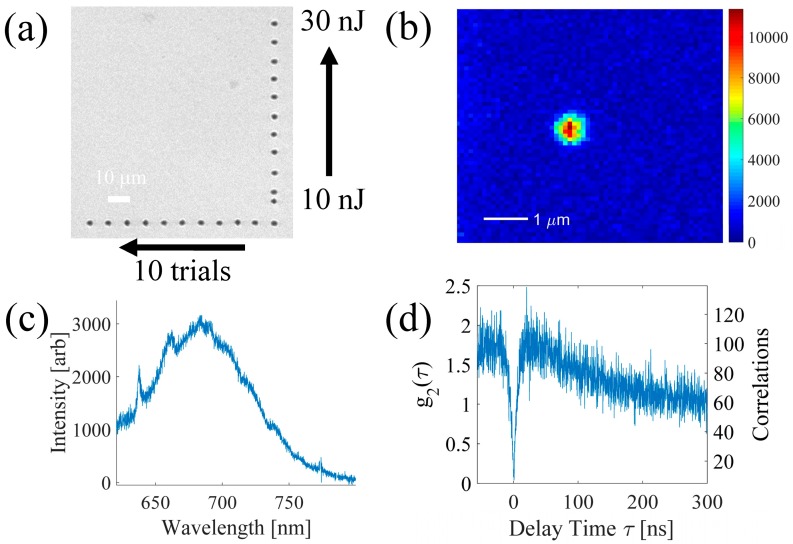
(**a**) Overhead microscope image of the static femtosecond laser exposure area. The black marker spots are written with higher energy and multiple laser pulses to identify the positions of the lower energy single static laser exposures which are not visible. (**b**) Overhead photoluminescence intensity map and (**c**) spectrum for single NV center produced by focused femtosecond laser pulse (24 nJ pulse energy) followed by annealing. (**d**) Intensity autocorrelation (corrected for background on left *y*-axis [23], raw uncorrected correlations counts on right *y*-axis) revealing single photon emission.

To produce vacancies at the desired position, we used single femtosecond laser pulses focused 25 µm below the surface with energies between 10 and 30 nJ, well below the amorphization threshold (~50 nJ). The regions selected for laser processing contained no pre-existing NV centers. For each pulse energy, 10 trials were performed. After the laser exposure, no visible modification was observed, as shown in Figure 5a. The sample was then annealed at 1000 °C for 3 h in a nitrogen atmosphere to avoid oxidation at the diamond surface. This temperature was selected based on previous studies that have shown that high annealing temperatures improve the properties of the resulting NV centers [8,21,22]. 

After annealing, the photoluminescence emission from non-irradiated diamond was the same as before the treatment. However, a photoluminescence (PL) signal showing an NV signature was found at the static laser exposure conditions, for pulse energies of 16 and 30 nJ. Below 16 nJ pulse energy, no PL signal was detected between 630 to 800 nm. In the energy range where NVs are formed, we performed cross correlation measurements, which revealed that pulse energies between 20 and 26 nJ had the highest probability of producing single NV centers. In particular, for 24 nJ, in our best sample, we found the probability of creating single NVs to be 80% (4 out of 5 trials), although this value is typically approximately 50% (average of five samples), similar to the success probability reported by Chen et al. [8]. The maximum of the NV emission is always located inside a 1 µm radius around the center of the static exposure. 

In Figure 5b–d, we present the results for an NV obtained with a 24 nJ static femtosecond laser pulse exposure. In Figure 5b, the overhead photoluminescence intensity map shows that the emission is well localized in a 1 µm^2^ area near the static exposure. The spectrum recorded at room temperature for this NV is shown in Figure 5c, where the characteristic ZPL at 637 nm and the broadband phonon side band are clearly visible. An intensity autocorrelation measurement of the photons detected is shown in Figure 5d, showing an antibunching dip with *g*^(2)^(0) well below 0.5, characteristic of single photon source emission. From these results, we can conclude than it is possible to create single NV centers with submicron precision using a single femtosecond laser without the use of beam shaping techniques. Future work will seek to integrate optical circuits and NV centers in diamond, all formed using femtosecond laser microfabrication. 

## 4. Conclusions 

In summary, we have demonstrated that femtosecond laser microfabrication can inscribe buried and single mode optical waveguides in diamond using type II geometry. The waveguides can be tuned to operate between 532 and 1550 nm wavelength by increasing the separation from 13 to 19 µm between the modification lines. The highest repetition rate of 500 kHz was found to produce less graphite in the modification lines and a higher quality guiding region, leading to lower damping losses. However, more work is required to better understand the mechanisms for waveguiding in diamond. We demonstrated the possibility of forming single NV centers in the bulk of diamond without the use of special beam shaping methods. The laser-written NV centers and optical circuits will serve as the building blocks for a diamond photonics platform that could enable both quantum information systems with optically connected entangled qubits and ultrasensitive and high-resolution optical magnetometers.
